# Genome-wide association study of the loci and candidate genes associated with agronomic traits in *Amomum villosum* Lour

**DOI:** 10.1371/journal.pone.0306806

**Published:** 2024-08-05

**Authors:** Wenxiu Li, Ping Luo, Yunfeng Shi, Hualin Zhang, Qing Yan, Yana Ye, Yanli Yao, Junjun He

**Affiliations:** 1 Chinese Academy of Tropical Agricultural Sciences/Zhanjiang Rubber Forest Economic Engineering Technology Research Center, Zhanjiang Experimental Station, Zhanjiang, Guangdong, China; 2 Huazhong Agricultural University, Wuhan, Hubei, China; 3 Chinese Academy of Tropical Agricultural Science/Key Laboratory of Tropical Fruit Biology, Ministry of Agriculture & Rural Affairs, Key Laboratory of Hainan Province for Postharvest Physiology and Technology of Tropical Horticultural Products, South Subtropical Crop Research Institute, Zhanjiang, Guangdong, China; University of Delhi, INDIA

## Abstract

*Amomum villosum* Lour. (*A*. *villosum*) is a valuable herbaceous plant that produces the famous traditional Chinese medicine Amori Fructus. Identifying molecular markers associated with the growth of *A*. *villosum* can facilitate molecular marker-assisted breeding of the plant. This study employed 75 *A*. *villosum* accessions as the test material and utilized 71 pairs of polymorphic simple sequence repeat (SSR) molecular markers to genotype the population. The study analyzed the association between SSR markers and phenotypic traits through the linkage imbalance and population structure analysis. Candidate genes associated with the molecular markers were also identified. The results showed that the phenotypic diversity index range of the 12 agronomic traits was 4.081–4.312 and conformed to a normal distribution. Moreover, 293 allelic variations were detected in the 75 accessions, with an average of 5.32 amplified alleles per loci, ranging from 3 to 8. The maximum number of amplified alleles for AVL12 was 8. The population structure and cluster analysis indicated that the accessions could be divided into two subgroups. Using the mixed linear model (MLM) model of population structure (Q)+kinship matrix (K) for association analysis, three SSR molecular markers significantly associated with the agronomic traits were detected. Fluorescence quantification was used to analyze the expression levels of six candidate genes, and it was found that three of the genes were differentially expressed in phenotypically different accessions. This study is the first to use SSR markers for genome-wide association study (GWAS) mapping and identification of the associated agronomic traits in *A*. *villosum*. The results of this study provide a basis for identifying genetic markers for growth traits for marker-assisted breeding in *A*. *villosum*.

## Introduction

*Wurfbainia villosa* (2n = 48), also known as *Amomum villosum* Lour., is a perennial plant belonging to the monophyletic genus *Wurfbainia* of the family Zingiberaceae [1]. *Amomi Fructus*, the dry and mature fruit of *A*. *villosum*, is one of the famous Chinese traditional medicines [2], with the potential to prevent miscarriage, warm the spleen and eliminate dampness [3]. *A*. *villosum* has been cultivated for over 1300 years in China [4] and currently has many accessions from different regions with significant phenotypic differences [5]. Evaluating these accessions and their genetic background is beneficial for improving the utilization rate, selection efficiency, and selective breeding of *A*. *villosum* [6]. Additionally, studying the correspondence between phenotypes and genotypes is crucial for marker-assisted breeding (MAS) [7].

Genetic diversity research is important for accession evaluation, core germplasm bank construction, and hybrid breeding of new varieties [8]. The most direct method for evaluating genetic diversity in plants involves morphological observation, but morphological observation is easily influenced by the environment. Therefore, genetic diversity evaluation often combines morphological observation with molecular marker technology to evaluate germplasm resources. Simple sequence repeats (SSRs) are common and important molecular markers with high polymorphism and repeatability [9]. SSRs have been widely used in genetic diversity [10] and association analysis-related research [11]. Genetic diversity analysis makes it possible to understand the genetic distance and variation between different accessions [12]. Genetic diversity analysis also helps eliminate accessions with high similarity based on cluster analysis results to construct a core germplasm bank while preserving as much genetic information as possible within the population [13]. In addition, it is necessary to select distant accessions or germplasm for hybridization to fully utilize heterosis in breeding. The genetic relationships between different accessions can be understood [14], and suitable parental materials can be easily selected for breeding [15] through genetic diversity analysis, thereby reducing the selection of parental combinations with similar genetic information and the breeding workload. Thus, genetic diversity analysis plays an important role in the selection of parental material for combinations [12]. Several studies have shown that the accessions of *A*. *villosum* are abundant and have significant differences in their main chemical components, content, and quality in different growing areas [5, 16, 17]. However, the number of collected and studied *A*. *villosum* accessions is relatively limited (currently up to 29) [17], and there is no detailed evaluation and identification of their growth traits.

Association analysis is an effective method for identifying genetic markers associated with quantitative traits [18]. Genome-wide association study (GWAS) is suitable for detecting the relationship between molecular markers or candidate genes and target traits in natural populations based on linkage disequilibrium (LD) [19]. GWAS can directly detect the relationship between phenotypes and genotypes in large populations without the need to construct hypotheses between phenotypes and genes, thus reducing the analysis cost at the genomic level for large samples [20]. The technique was first applied in plants in 2001 when Hansen et al. [19] conducted research on Sea beet. Several researchers subsequently utilized SSRs in plants, such as maize [21, 22] and wheat [11], to identify the molecular markers closely linked to the target traits. Although GWAS has been widely used in plant research, there are no reports on its usage on *A*. *villosum*. Studies have shown that growth traits (such as leaf width, ligule length, etc.) of *A*. *villosum* are closely related to the number of capsules [23]. Therefore, identifying genetic markers associated with these growth traits would provide a reference basis for breeding high-yielding *A*. *villosum* varieties.

This study employed 75 accessions of *A*. *villosum* Lour. (which are currently the most reported) as the test material and utilized 71 pairs of polymorphic SSR molecular markers to genotype the population. The study analyzed the association between SSR markers and phenotypic traits through the linkage imbalance and population structure analysis. The candidate genes were selected based on linkage markers, and fluorescence quantitative expression pattern analysis was conducted, laying the foundation for genetic research and molecular marker-assisted breeding of complex quantitative traits in *A*. *villosum*.

## Materials and methods

### Plant material

A natural population of 75 *A*. *villosum* accessions were collected from Guangdong (31 samples), Guangxi (20 samples), Yunnan (22 samples), Fujian (1 samples) and Hainan (1 samples) provinces in China (S1 File). After collection, the accessions were intercropped in shaded areas under the rubber forest. Due to the asexual reproduction ability of *A*. *villosum*, many new shoots were produced in each material after planting for a period of time. Ten seedlings with relatively consistent sizes were randomly selected from each material for planting. The experiment was conducted in a greenhouse, each plot measuring 2 × 5 meters and containing 10 plants. The experimental site was located in Zhanjiang City, with a geographical location of 110 a geographical location of and an elevation of about 21 meters. The region had a tropical northern monsoon climate, with an annual average temperature of 23°C, precipitation of 1200 mm–1800 mm, sunshine hours of 1700 h–2100 h, annual average total radiation of 175 W/m^2^, and average light intensity of about 897Lx. The soil type was laterite.

The germplasm utilized in this study was grown in the germplasm resource garden situated at No. 1 Huxiu Road, Guangdong Province, China. The garden is affiliated with the Zhanjiang Experimental Station of the Chinese Academy of Tropical Agricultural Sciences. We obtained the permissions (verbal consent) and can confirm no conflicting interests.

### Phenotyping

After one year of planting, five plants were randomly selected from each test plot for investigation, according to the "*Amomum villosum* Lour." section of the "Specification for the Description of Germplasm Resources of Southern Medicines" [24] and the Chinese agricultural industry standard "Guidelines for Plant Variety Specificity (Distinguishability), Consistency, and Stability Testing *Amomum villosum* Lour." [25]. Twelve agronomic traits were investigated, including the plant height (PH), stem diameter (SD), number of blades (NB), number of ramets (NR), leaf length (LL), leaf width (LW), ligule length (LEL), total length of stolons (TLS), stolon thickness (ST), stolon internode length (SIL), number of new shoots (NNS), and total number of ramets (TNR). PH, LL, LW, LEL, TLS, and SIL were measured using a ruler, while LL, LW, and LEL were measured using the third leaf blade from the top. SD and ST were measured at the breast height using vernier calipers. The average value of each trait was used for subsequent analysis.

### DNA extraction

The DNA was extracted using a DP305 plant DNA kit (TIANGEN Biotech Co., Ltd., Beijing, China) [16]. The quality and concentration of the DNA were determined by agarose gel electrophoresis and NanoDrop2000 ultra-micro ultraviolet spectrophotometer (Thermo Scientific, MIT, USA) [26], and the DNA was stored at -20°C for subsequent analysis.

### Primer screening, PCR amplification and sequencing

A total of 71 SSR primer pairs were used, of which 63 pairs were obtained from the transcriptome data analysis of *A*. *villosum* developed in our laboratory (at Zhanjiang Experimental Station, Chinese Academy of Tropical Agricultural Sciences), and 8 were obtained from a previous study [27] (S1 File). The SSR primers were synthesized by Shanghai Shenggong Biotechnology Co., Ltd. SSR markers were screened using five morphologically distinct germplasm, including S8, S10, S40, S52, and S92, to identify polymorphic markers and those with good amplification effects were selected. Subsequently, 55 pairs of SSR markers were selected for genotyping the 75 accessions of *A*. *villosum* using PCR amplification.

The PCR reaction volume was 10 μl, consisting of 5 μl of 2x Taq plus Master Mix (Bishsarp Life Sciences Co., Ltd, China), 0.5 μl of the forward and reverse primers (each 0.5 μM), 1 μl of genomic DNA (10ng), and 3 μl of double distilled water (ddH_2_O). The PCR program setting and PCR product analysis were conducted according to the method by Yeboah et al. [26].

### Data analysis

#### Phenotypic data analyses

Descriptive statistics, viz. minimum, maximum, mean, range, and standard deviation values, were calculated using SPSS version 21.0, while the coefficient of variation (CV) and diversity index (*H*_*e*_’) were calculated using EXCEL 2021 [28]. Pearson’s correlation analysis of the different traits was conducted using the *corrplot* package in R 3.4.5 [29].

#### Genotypic data analysis

Each amplification product was considered a unit character, with the SSR-PCR bands being scored as either present (1) or absent (0) and missing bands recorded as 9. Various parameters related to marker polymorphism, such as major allele frequency (MAF), the number of alleles, the number of genotypes, gene diversity, and the polymorphism information content (PIC), were calculated using Powermaker V3.25 [30].

#### Population structure, relative kinship, LD, marker-trait association, and mining of beneficial alleles

Population structure (Q matrix) and a kinship coefficient estimation matrix (K) of the tested *A*. *villosum* accessions were analyzed using the STRUCTURE 2.3.4 software [13] and SPAGeDi software [31], respectively, as previously described [26]. LD, genome-wide association mapping, manhattan plot, and quantile-quantile (QQ) plots were generated using TASSEL 3.0.174 software [32] according to the Bonferroni test threshold [33, 34], which was set as 0.05/total SSR makers (P<0.05/293 = 1.6 × 10^−4^). To calculate the coefficient of genetic differentiation (FST) and gene flow (Nm), use Excel and GenAlEx 6.5 (Due to the small sample size in Hainan and Fujian, no statistics were conducted).

#### Candidate gene prediction and expression analyses

Based on the GWAS analysis results, functionally annotated genes were searched within the 200 Kb interval upstream and downstream of the molecular markers in the *A*. *villosum* genome [1, 9]. The candidate genes related to traits were selected based on the annotation results, and the identified regions were subjected to BLAST analysis using BlastP on the National Center for Biotechnology Information (NCBI) website to predict potential candidate genes. The fresh roots stems and leaves of the plant materials with significantly different LEL and NNS were selected for quantitative fluorescence expression pattern analysis. Total RNA was extracted using FastPure® Tissue Total RNA Isolation Kit (Vazyme #RC101, China). The oligo-(dT)18 primer and 1 μg of total RNA were used to synthesize cDNA using the HiScript® III RT SuperMix for qPCR (+gDNA wiper)(Vazyme #R323, China). The sequences of candidate genes were retrieved from the NCBI database (https://doi.org/10.6084/m9.figshare.19200005.v1), and the primers targeting these genes were designed using the qPCR 2 Primers Intercalating Dyes tool (https://sg.idtdna.com/pages) and were synthesized by Sangon Biotechnology Co., Ltd. Quantitative reverse-transcription polymerase chain reaction (qRT-PCR) was performed on LightCycler480 II using ChamQTM Universal SYBR® qPCR Master Mix (Vazyme #Q711, China). The reaction conditions were 95°C for 2 min, followed by 45 cycles of 95°C for 20 s, 55°C for 15 s, and 72°C for 30 s. The *TUA* gene was selected as the endogenous control, and the relative gene expression was measured using the delta-delta Ct (2^–ΔΔCt^) method. The data were analyzed in Microsoft Excel 2013 and compiled in GraphPad Prism.

## Results

### Phenotypic characterization

**Fig 1:**
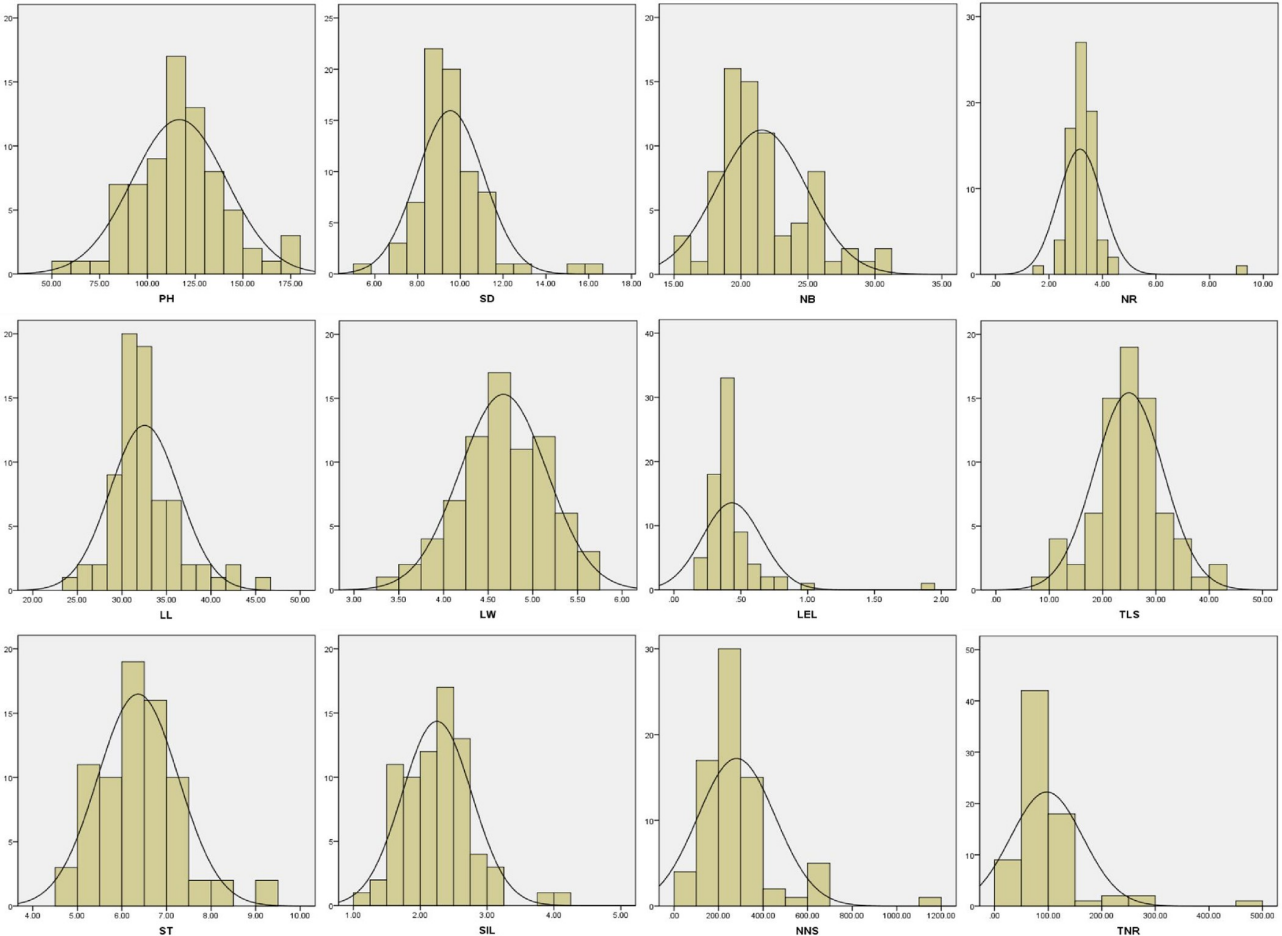
Frequency distribution of the 12 agronomic traits of *Amomum villosum* Lour. PH **=** height; SD **=** stem diameter; NB **=** number of blades; NR **=** number of ramets; LL **=** leaf Length; LW **=** leaf width; LEL **=** ligule length; TLS **=** total length of stolons; ST **=** stolon thickness; SIL **=** stolon internode length; NNS **=** number of new shoots; TNR **=** total number of ramets.

The Shannon index (*H*_*e*_*’*) reflects species richness and evenness. The more uniform the number of individuals, the greater the *H*_*e*_*’* and the smaller the degree of variation. Conversely, the coefficient of variation reflects the opposite result, i.e., the larger the coefficient of variation, the greater the degree of dispersion. Statistical analysis showed that the *H*_*e*_*’* values of the 12 phenotypic traits of *A*. *villosum* accessions had a range of 4.146–4.312 (Table 1), and those of SD, NB, NR, LL, LW, and ST were higher than 4.3. The *H*_*e*_*’* of NR was the lowest at 4.146, while that of LW was the highest at 4.312. The maximum coefficient of variation for NR was 0.691, and the minimum coefficient of variation for LW was 0.105. Overall, these results indicate that NR had the highest variation, while LW had the lowest. As shown in Fig 1, the frequency distribution of the 9 phenotypic traits conformed to normal distribution.

**Table 1:** Diversity analysis of the growth traits of *Amomum villosum* Lour.

Trait^(a)^	Minimum	Maximum	Mean	Range	SD^(b)^	CV^(c)^	*H*_*e*_’^(d)^	K-S^(e)^
PH (cm)	52.58	177.94	116.72	125.36	24.80	0.212	4.295	0.941
SD/(mm)	5.60	12.78	9.33	7.18	1.21	0.130	4.309	0.000
NB	15.00	30.60	21.55	15.60	3.33	0.154	4.306	0.164
NR	1.60	4.40	3.08	2.80	0.45	0.147	4.307	0.000
LL/(cm)	23.78	46.04	32.55	22.26	3.88	0.119	4.311	0.111
LW/(cm)	3.48	5.74	4.67	2.26	0.49	0.105	4.312	0.986
LEL/(cm)	0.20	1.02	0.42	0.82	0.14	0.346	4.265	0.001
TLS/(cm)	7.48	42.80	24.94	35.32	6.46	0.259	4.283	0.878
ST/(mm)	4.58	9.15	6.36	4.58	0.91	0.143	4.308	0.857
SIL/(cm)	0.20	1.88	0.43	1.68	0.22	0.510	4.227	0.720
NNS	21.00	491.00	97.35	470.00	67.28	0.691	4.146	0.070
TNR	48.00	1148.00	281.21	1100.00	173.60	0.617	4.163	0.060

^(a)^ PH **=** plant height; SD **=** stem diameter; NB **=** number of blades; NR **=** number of ramet; LL **=** leaf Length; LW **=** leaf width; LEL **=** ligule length; TLS **=** total length of stolons; ST **=** stolon thicknes; SIL **=** stolon internode length; NNS **=** number of new shoots; TNR **=** total number of ramets.

^(b)^ Standard deviation.

^(c)^ Coefficient of variation.

^(d)^ Diversity index.

^(e)^ Kolmogorov-Smirnov test, ‘>0.05’ indicates normal distribution; Extreme maximum values were removed during NNS and TNR statistics.

### Correlation analyses of the phenotypic traits

**Fig 2:**
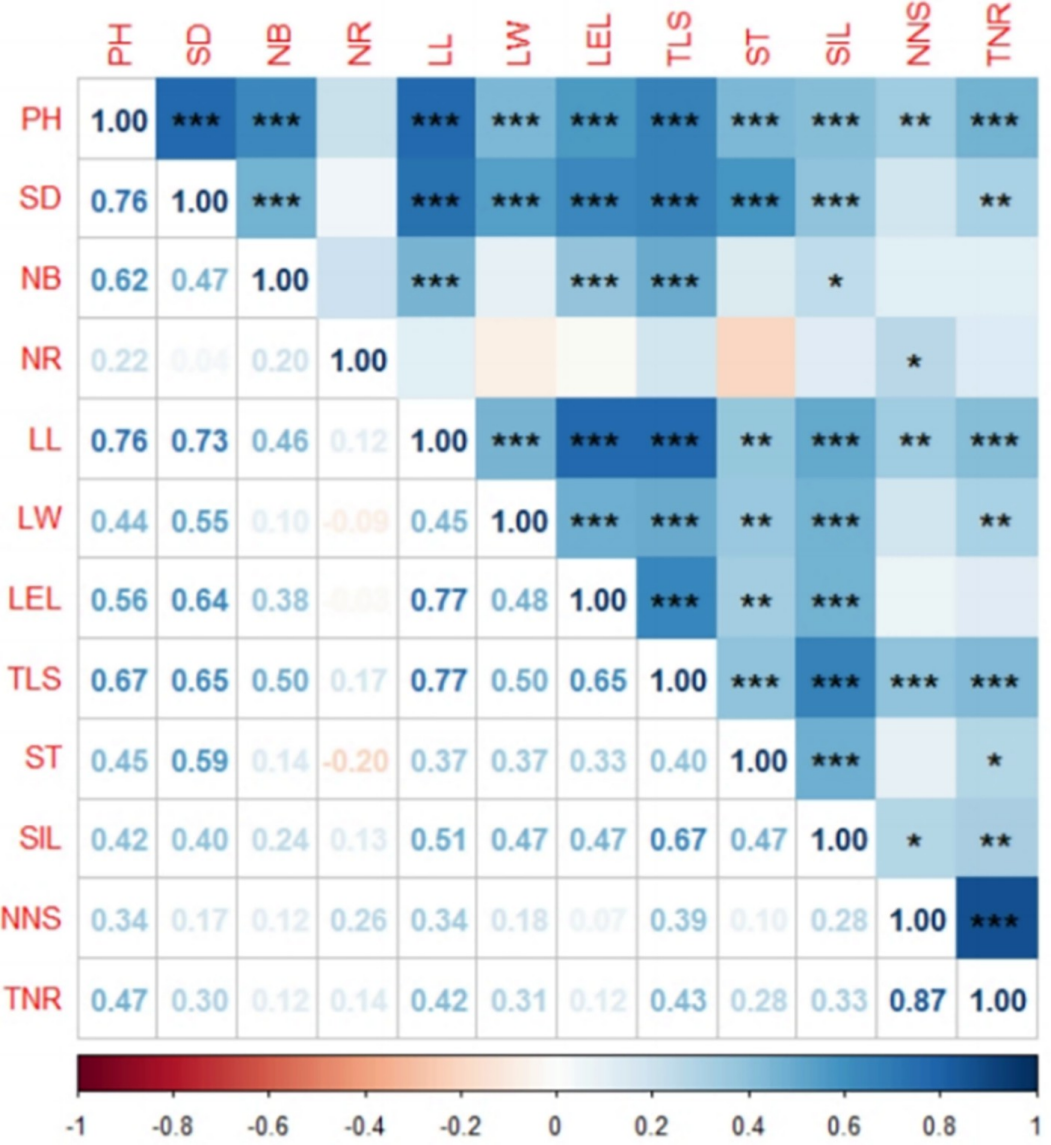
Correlation heat map of the 12 agronomic traits of *Amomum villosum* Lour. ‘*’ indicates correlation significance at the 0.05 level, while ‘**’ represents significance at the 0.01 level and ‘***’ shows significance at the 0.001 level. PH **=** height; SD **=** stem diameter; NB **=** number of blades; NR **=** number of ramets; LL **=** leaf Length; LW **=** leaf width; LEL **=** ligule length; TLS **=** total length of stolons; ST **=** stolon thickness; SIL **=** stolon internode length; NNS **=** number of new shoots; TNR **=** total number of ramets.

**Fig 3:**
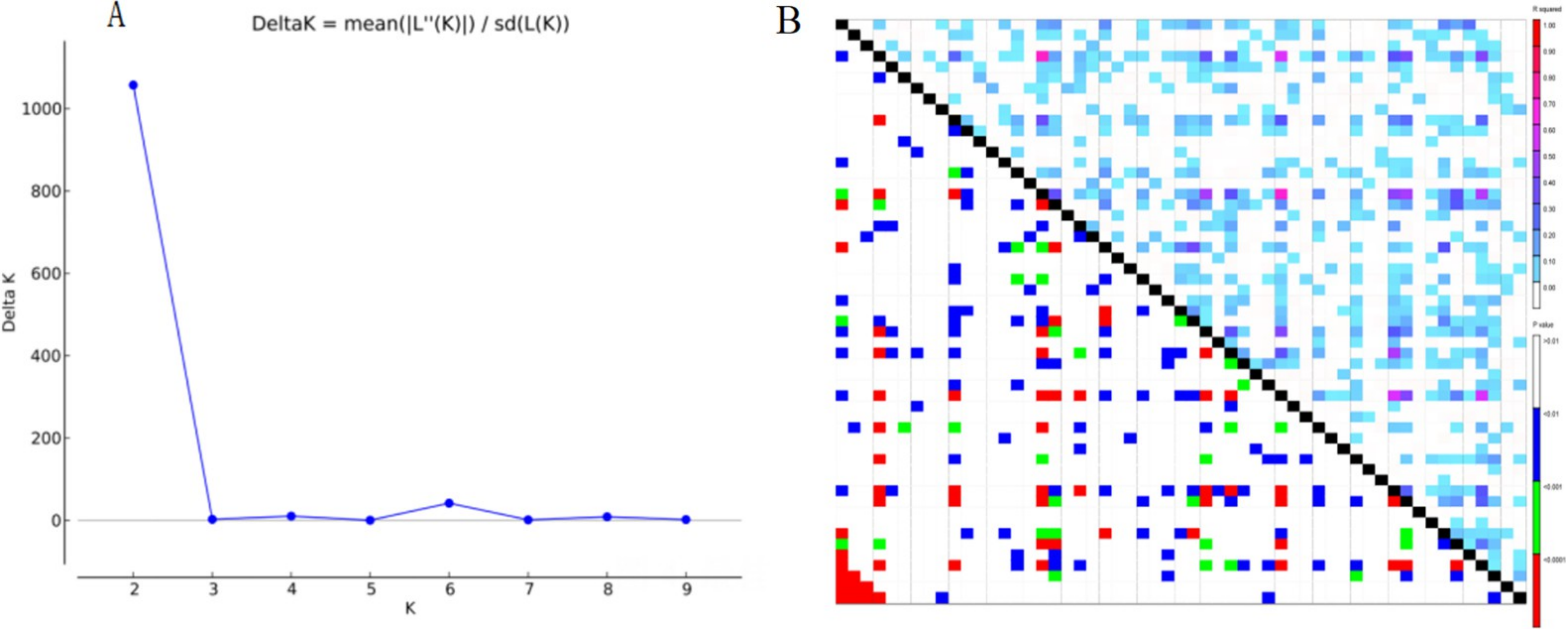
Population structure (A) and cluster analysis (B) of the 75 *Amomum villosum* accessions.

The correlation analysis of the 12 traits was conducted, and the correlation coefficients are shown in Fig 3. The results indicate that PH had a highly significant positive correlation with SD, NB, LL, LW, LEL, TLS, ST, SIL, and TNR at the P < 0.001 significance level and with NNS at P < 0.01 significance level (Fig 2). Additionally, SD had a highly significant positive correlation with NB, LL, LW, LEL, TLS, ST, and SIL (P < 0.001) and a significant positive correlation with TNR (P < 0.01). NB was not correlated with NR, LW, ST, NNS, and TNR but had a highly significant positive correlation with LL, LEL, and TLS (P < 0.001) and a significant positive correlation with SIL (P < 0.05). NR only had a significant positive correlation with NNS at the P < 0.05 significance level. Furthermore, LL exhibited a highly significant positive correlation with LW, LEL, SIL, and TNR at the P < 0.001 significance level and a highly significant positive correlation with ST and NNS at the P < 0.01 significance level. Similarly, LW demonstrated a highly significant positive correlation with LEL, TLS, and SIL at the P < 0.001 significance level and a highly significant positive correlation with ST and TNR at the P < 0.01 significance level. LEL showed a highly significant positive correlation with TLS and SIL at the P < 0.001 significance level and a highly significant positive correlation with ST at the P < 0.01 significance level. Furthermore, a highly significant positive correlation existed between TLS and the ST, SIL, NNS, and TNR (P < 0.001). ST exhibited a highly significant positive correlation with SIL (P < 0.001) and a significant positive correlation with TNR (P < 0.05). There was also a highly significant positive correlation between SIL and TNR (P < 0.01) and a significant positive correlation between SIL and NNS (P < 0.05).

### Population structure and cluster analysis

**Fig 4:**
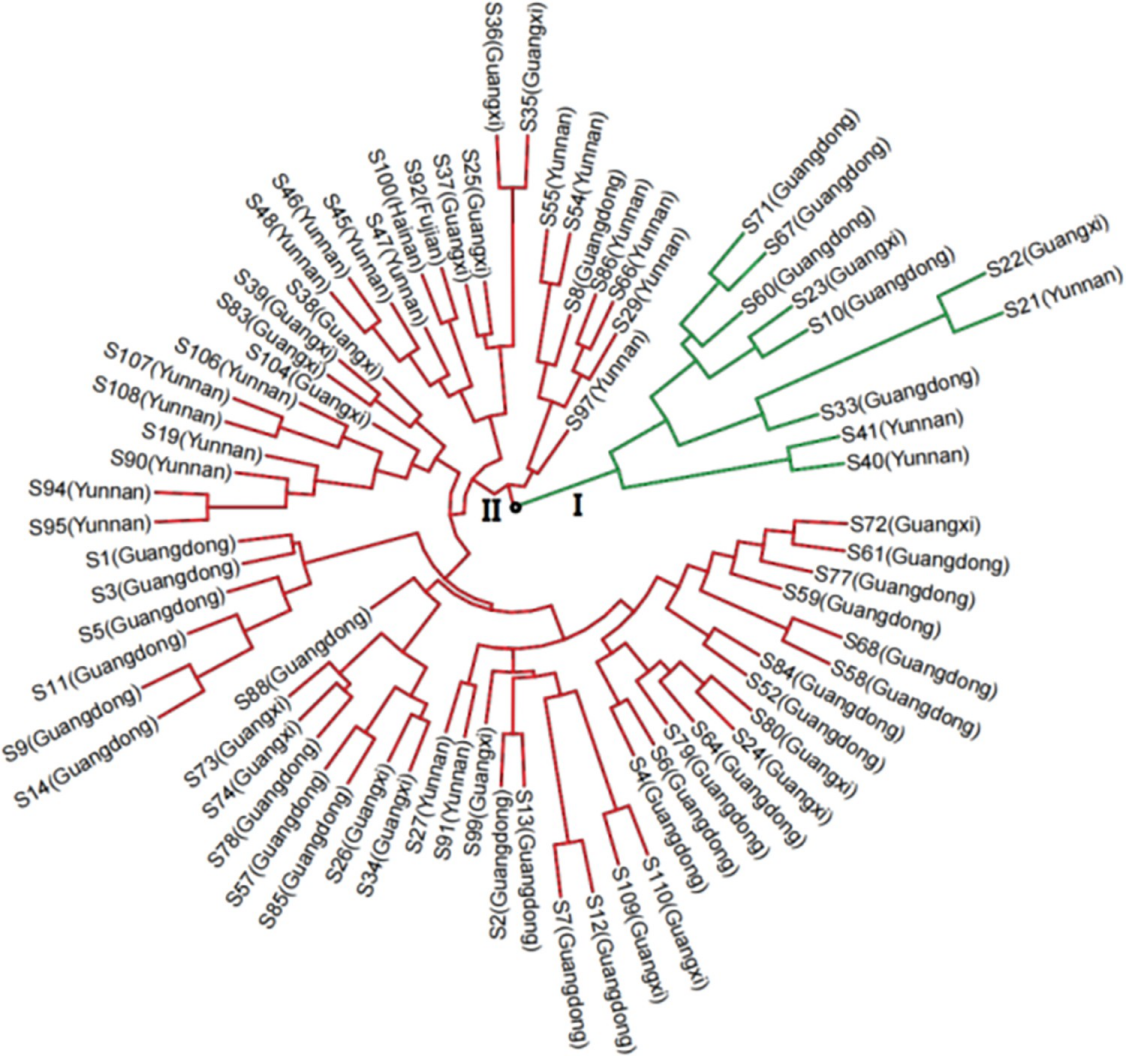
The linkage disequilibrium (LD) distribution among 55 simple sequence repeat (SSR) loci in 75 *Amomum villosum* accessions.

The population structure and cluster analysis indicated that the *A*. *villosum* accessions could be categorized into two major subpopulations. The group population structure analyzed through the “Structure Harvester” program showed a sharp peak of the *ad hoc* quantity (*ΔK*) at K = 2 (Fig 3A). Cluster analysis was performed using the RStudio software, and the clustering tree showed the most likely genetic relationship between the *A*. *villosum* accessions (Fig 4). The cluster analysis categorized all materials into two major clusters, with the first cluster containing 10 materials and the second 65. Cluster I comprised two branches, one containing two materials from Yunnan and the other having eight materials from Guangdong, Guangxi, and Yunnan. This branch indicated a closer genetic relationship among Guangdong, Guangxi, and Yunnan materials. Cluster two also had two major branches, with one consisting of 7 materials from Yunnan and the other encompassing 58 materials. Overall, the cluster analysis results showed that the *A*. *villosum* accessions from different places in China were dependent but were not significantly correlated with their origin, indicating that though the accessions were from different sources, they were not significantly different.

The analysis of the FST and Nm yielded the following results in the Table 2 below. The coefficient of FST between populations ranges from 0.016 to 0.027, with the lowest FST between populations A and B (0.016) and the highest FST between populations B and C (0.027). However, all FST values are less than 0.05, indicating a low degree of genetic differentiation between populations. In terms of Nm, the highest Nm is observed between populations A and B, at 15.335, suggesting high genetic similarity. Conversely, the lowest Nm is found between populations B and C, at 8.6, indicative of low genetic similarity. Selecting these two populations (B and C) for hybrid breeding is more likely to yield new variations.

**Table 2:** Values of FST (below diagonal) and Nm (above diagonal) among 3 populations of *Amomum villosum* Lour.

Population	A	B	C
A		15.335	9.398
B	0.016		8.896
C	0.026	0.027	

Note: A = population of Guangdong; B = population of Guangxi; C = population of Yunnan

The degree of LD was evaluated using the average coefficient of determination (R^2^) at 0.01 (Fig 3B). The distribution analysis of LD showed that 13.06% and 6.32% of R^2^ were statistically significant at *P* < 0.01 and *P* < 0.001, respectively.

### Marker-trait association

**Fig 5:**
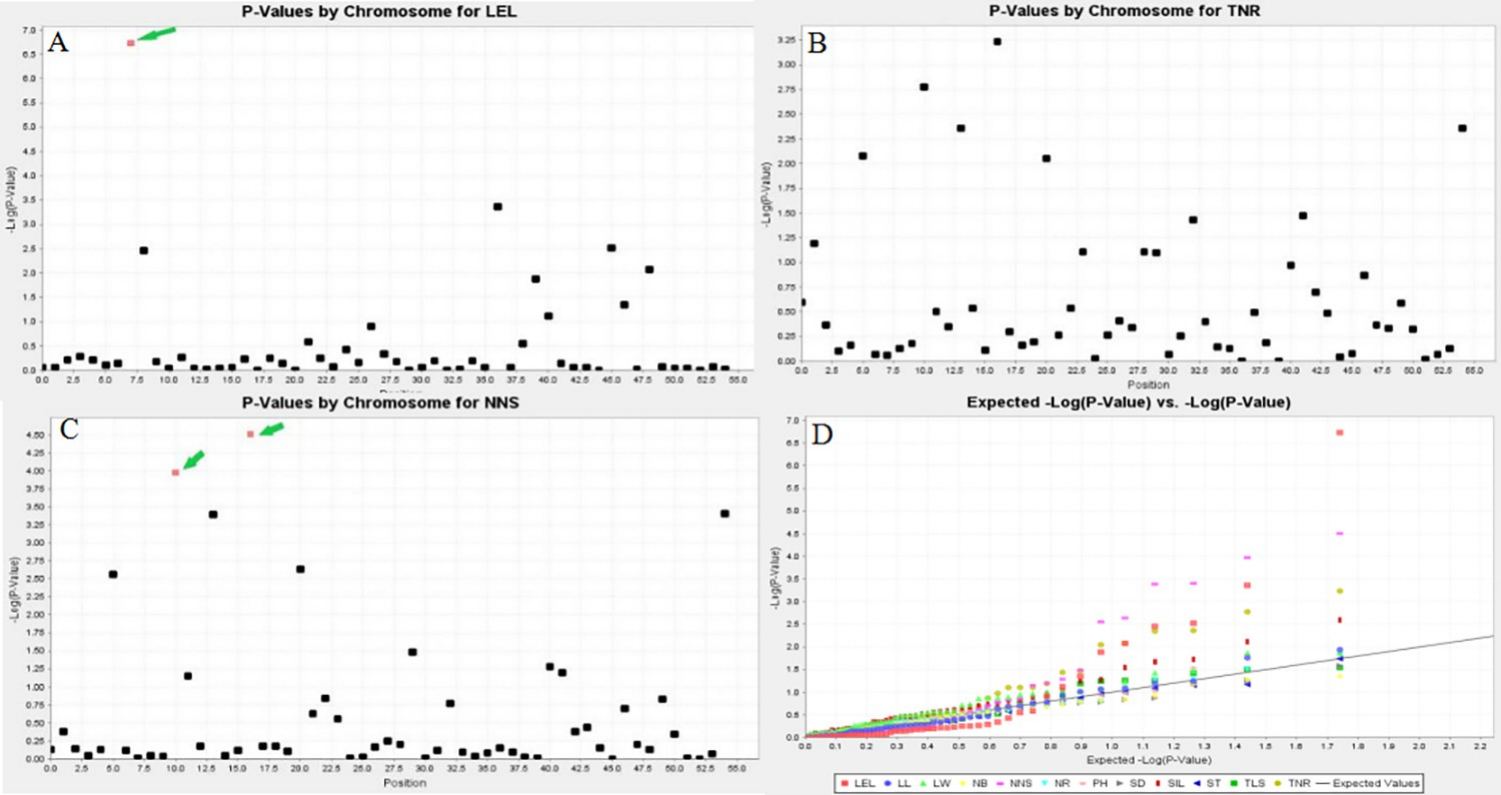
Genome-wide association analysis of the growth traits of *Amomum villosum* Lour. using a mixed linear model. Manhattan plot of (A) ligule length, (B) total number of ramets and (C) number of new shoots. (D) Quantile-quantile (QQ) plot of all the 12 traits.

The association between the SSR markers and the traits was analyzed using a mixed linear model (MLM). Three significant SSR markers were found to be associated with growth traits in *A*. *villosum* (Fig 5A and 5C). NNS was linked to two makers, AVL14 and AVL22, which had the phenotypic variance explained (PVE) proportions of 41.31% and 38.07%, respectively. The other marker (AVL11) was associated with LEL and had a PVE value of 60.33%. The QQ and Manhattan plots for the maker-trait associations are shown in Fig 5D. There was evident statistic inflation for LEL and NNS. Based on the physical location of the molecular marker sites associated with the GWAS analysis, six candidate genes with functional annotation were identified within the 200 Kb interval upstream and downstream of the molecular markers in the *A*. *villosum* genome (Table 3).

**Table 3:** Simple sequence repeat (SSR) markers associated with target traits (MLM) of *Amomum villosum* Lour.

Trait	Marker	F-value	P-value	Phenotypic variance explained (%)	Chromosome	Position (bp)	Candidate gene ID	Description
LEL	AVL11	14.64	1.86E-07	60.33	LG23	9730351>> —85—>>9730475	Wv_072909	LRR receptor kinase SERK2-like (LOC122018488)
							Wv_072917	oleosin
NNS	AVL22	9.30	3.10E-05	38.07	LG06	17632519>>—85—>>17632644	Wv_033832	BEL1-like homeodmain protein 4 (LOC121981163)
							Wv_033833	Histone-lysine N-methyltransferase SUVR5-like
NNS	AVL14	6.07	1.05E-04	41.31	LG06	53462795>>—111—>>53462946	Wv_038222	Alanine-tRNA ligase, chloroplastic/mitochondrial-like
							Wv_038224	uncharacterized

### Candidate genes and their relative expression in four different accessions

**Fig 6:**
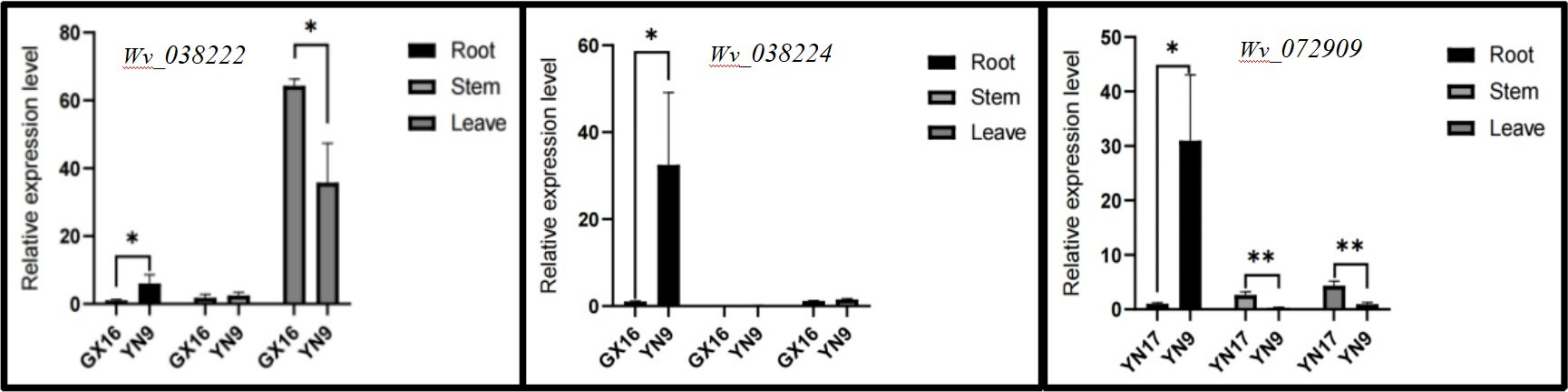
The expression levels of three candidate genes showing significant differences in different tissues of the phenotypically different accessions (GX16 with the most NNS, YN9 with the least NNS, YN17 with the longest LEL, and YN9 with the shortest LEL). Data represent the mean of three replicates. Significant differences are indicated as * *P* < 0.05 and ** *P* < 0.01. NNS **=** number of new shoots; LEL = ligule length.

The relative expression levels of six candidate genes in different tissues of *A*. *villosum* accessions (GX16 with the most NNS, YN9 with the least NNS, YN17 with the longest LEL, and YN9 with the shortest LEL) were determined using qRT-PCR. As shown in Fig 6, three candidate genes showed significant differences in different tissues of the four accessions. The relative expression level of *Wv_038222* significantly differed in the roots and leaves of GX16 and YN9, while that of *Wv_038224* significantly differed in the roots of GX16 and YN9. Moreover, *Wv_072909* showed highly significant differences in the roots, stems, and leaves of YN17 and YN9.

## Discussion

### Phenotypic characterization, population structure and LD analysis of the 75 *A*. *villosum* accessions

Descriptive statistics and correlation analysis reflect the differences and similarities in population phenotypic traits [35]. However, the magnitude of variation amplitude and coefficient of variation indicate the phenotypic richness in the population. A larger variation amplitude and coefficient of variation mean that the population has a high degree of trait variation and phenotypic diversity [36]. The diversity index of *A*. *villosum* accessions ranged from 4.081 to 4.312, with a coefficient of variation ranging from 0.105 to 0.691. This indicated that the *A*. *villosum* accessions collected from Yunnan, Guangxi, Guangdong, Fujian, and Hainan provinces in China were different, probably due to geographical isolation restrictions, which resulted in complex genetic components [37], leading to differences in the phenotypic traits of germplasm in different regions. Through correlation analysis, we found that most growth traits were correlated, indicating that they have a promoting effect on each other.

### Genetic diversity and LD analysis of the 75 *A*. *villosum* accessions

The population structure is an important factor affecting the association analysis results [36]. False-positive associations should be eliminated in cases where the population structure of the association is simple and conforms with the cluster analysis [26]. In this study, the population structure analysis showed that the *A*. *villosum* accessions were categorized into two subgroups. Similarly, the cluster analysis also grouped the accessions into two groups, indicating that *A*. *villosum* generally has a simple population structure, similar to the results of Li et al. [37] and Xu et al. [5]. Notably, *A*. *villosum* accessions from Guangdong province were categorized into different subgroups and had close genetic relationships with germplasm from different regions. This may be because Guangdong is the main production area of *A*. *villosum* and distributes germplasm resources to other regions, resulting in a high degree of similarity.

LD is the foundation of association analysis, and the degree of LD in the population determines the resolution of association analysis. Evaluating the whole genome LD before conducting GWAS simplifies the process of association analysis [38]. R^2^ is an indicator for measuring the degree of LD, with a range of 0–1. The closer the R^2^ is to 1, the closer the linkage. The average R^2^ obtained in this study was 0.099, which was higher than that reported in castor bean [26] and cotton [39] but lower than reported in soybean [34] and rice [40]. However, overall, the degree of LD in the *A*. *villosum* population was still low, probably because *A*. *villosum* is a cross-pollinated species with a higher recombination rate and LD break, resulting in a lower level of LD [39]. This lower level of LD results in weaker association signals and fewer associated marker loci. Therefore, if conditions permit, it is necessary to appropriately increase the number of markers to increase the strength of the associated signals.

### Marker-trait associations and mining of beneficial alleles in *A*. *villosum*

This study is the first to perform GWAS in *A*. *villosum*. Three SSR molecular markers (AVL11, AVL22 and AVL14) significantly associated with growth traits of *A*. *villosum* were detected using the MLM (Q+K) method. Two loci, AVL22 and AVL14, with a PVE of 38.73% and 42.27%, respectively, were linked to NNS, suggesting that a trait could be affected by multiple genes [41, 42]. Meanwhile, LEL was associated with the AVL11 locus with a PVE of 60.33%. The association results showed that 55 marker loci were related to growth traits, explaining 38.73% to 60.33%, which was higher than those reported in other crops, such as soybean (5.42–27.6%) [34], castor (7.32–20.47%) [26], and peanut (11.22–32.30%) [43]. This indicated that these loci are promising genetic markers that should be evaluated for use in MAS [44].

### Predicted putative candidate genes and their relative expression levels

Based on the expression analysis, the three genes exhibited significant differences in their expression levels across various tissues of different germplasms. Among them, *Wv-038222* and *Wv-038224* are potential candidates for the same marker AVL14 and are both associated with the number of new shoots in the plant. The primary factor influencing the number of new shoots is the meristematic ability of the root and stem. *Wv-038222* and *Wv-038224* had low expression in GX16 and high expression in YN9, indicating that high expression of these genes inhibits the growth of the root and stem, thereby decreasing the number of new shoots. Through CD-Search comparison in NCBI, it was discovered that *Wv-038222* shares similarities with the Alanine-tRNA ligase (*AlaRS*) gene. Previous studies indicated that the *AlaRS* gene is linked to embryonic development and functions as an enzyme in aminoacylation [45]. While the specific role of the *AlaRS* gene in root and stem differentiation remains unknown, mutations in *AlaRS* gene expression can result in cell or embryo death, thus impacting plant growth and development. Hence, it can be inferred that the *Wv-038222* gene may play a certain role in the meristematic ability of the root and stem tissues in *A*. *villosum*.

Furthermore, *Wv-038222* was also characterized by significantly higher expression levels in YN9 leaves than GX16. However, there are no comparison data on the *Wv-038224* gene in the NCBI database, and the action mechanism of the gene requires further research.

The *Wv*.*072909* gene is associated with ligule length. The expression level of the gene in the root of YN17 was significantly lower than that of YN9, while the expression level in the stem and leaves of YN17 was notably higher than that of YN9. This suggests that the high expression in the leaves and stems promotes leaf ligule elongation, whereas high expression in the root system has no effect. Upon comparison, it was discovered that the *Wv-072909* gene is similar to the FRQ1 superfamily member COG5126, identified as a Ca2+-binding protein gene. Research indicates that this gene may be closely linked to plant stress resistance [46]. The leaf ligule of *A*. *villosum* resembles that of cereal crops in the Poaceae family, containing numerous stomata that effectively reduce water evaporation. For example, rice maintains water in intense ultraviolet environments through the stomata, thus enhancing its drought resistance. Additionally, these stomata can create a confined space, maintaining a relatively stable temperature and resisting the intrusion of cold weather. Therefore, the presence of leaf ligule in *A*. *villosum* may hold a significance akin to that of cereal crops. It can be inferred that the expression of *Wv-072909* influenced the leaf length of *A*. *villosum*, thereby impacting the stress resistance of *A*. *villosum*.

In summary, the three differentially expressed genes may be candidate genes associated with the number of new shoots and ligule length of *A*. *villosum*. However, it is necessary to verify these candidate genes through transgenic validation to accurately obtain their functions.

## Conclusion

In summary, the 12 agronomic traits of *A*. *villosum* germplasm resources exhibited a normal distribution, indicating that these traits are mainly controlled by multiple genes. We detected 293 allelic variations using SSR primers, with an average amplification of 5.32 alleles and a variation range of 3–8, indicating that the SSR markers were feasible and highly polymorphic. Using the MLM model of Q+K for association analysis, three SSR molecular markers (AVL11, AVL22 and AVL14) significantly associated with the agronomic traits were detected. The genes on the left and right borders of the markers were selected as candidate genes, and their differential expression in different tissues was analyzed in four phenotypically different accessions. Finally, three related candidate genes were identified. The NNS was found to be associated with the AVL22 and AVL14 loci, while the LEL was linked to the AVL11 locus. This indicates the potential of these loci as genetic markers, and their application in MAS should be further validated.

## Supporting information

S1 File Supplementary table includes accessions, phenotypic, genotype, primer information, and coding sequences of the candidate genes. (XLSX)
